# Content Analysis of YouTube Videos That Demonstrate Panoramic Radiography

**DOI:** 10.3390/healthcare10061093

**Published:** 2022-06-13

**Authors:** Marlene Grillon, Andy Wai Kan Yeung

**Affiliations:** Oral and Maxillofacial Radiology, Applied Oral Sciences and Community Dental Care, Faculty of Dentistry, The University of Hong Kong, Hong Kong SAR, China; mgln@hku.hk

**Keywords:** YouTube video, dental education, oral and maxillofacial radiology, panoramic, student-centered learning

## Abstract

In this digital era, dental students often search for online resources for self-directed learning. YouTube is one of the most commonly sought online platforms for educational or instructional videos. No prior study has examined the validity of panoramic radiography videos available on YouTube. This study provides a content analysis of these YouTube videos. A search for relevant YouTube videos was conducted in April 2022. The search string was: (panoramic OR pan OR OPG) AND (dental OR dentistry OR X-ray). The first 100 videos that resulted from the search and their related videos were screened. Exclusion criteria included irrelevance (e.g., no demonstration of panoramic radiography procedures) and non-English videos. For each included video, the following parameters were recorded: image receptor type, patient age, patient type (real patient, animation, or phantom head), patient preparation procedures, machine preparation, patient positioning, and operator safety. The number of views, comments, likes, and channel subscribers were recorded, as well as the video duration and the age of the video. Forty videos were included and analyzed. Most of the videos demonstrated digital panoramic radiography with an adult patient. Procedures on the patient and machine preparations as well as patient positioning were generally explained well. However, most videos did not well-demonstrate operator safety details concerning the use of adequate personal protective equipment. View count, comment count, and channel subscriber count positively correlated with the like count. Clinicians and students should carefully critique the content of such instructional videos and refer to the contents from other sources such as user manuals and latest recommendations from local authorities.

## 1. Introduction

Panoramic radiography is an essential part of modern dentistry. Taking a panoramic radiograph as part of a “routine screening” for every new patient is controversial and may be considered unnecessary [1]. However, it was reported that as many as nearly 42% of general dentists adopted such practice in the United Kingdom [2]. A panoramic radiograph visualizes a patient’s dental and maxillofacial regions and provides additional morphological and pathological information not available from intraoral radiographs [3,4]. However, such information can only be provided from a clear image taken correctly. To obtain good image quality with a correct workflow, clinicians and dental students can refer to textbooks and user manuals to familiarize themselves to the radiographic procedures. Customized instructional videos would be ideal for such purposes [5,6,7]. However, such videos may not always be available to the general dental community. YouTube videos could be fit for learning new dental procedures or refreshing the memories on previously learnt procedures, such as for endodontic and oral surgical treatments [8,9,10]. Undoubtedly, there are multiple educational healthcare video sources across the internet, such as YouTube, Virtual Derm Surg, Procedures Consult, MedClip, ORLive, AccessSurgery, MedlinePlus, and Medical Videos [11]. Nonetheless, students and clinicians have already been widely consulting YouTube as a learning tool, such that it is the most frequently used educational video source for anatomical knowledge [12] and medical surgery preparation [13]. Being open access and having non-peer-reviewed user-contributed contents, YouTube contains very good materials for healthcare education, but also potentially much misinformation/inaccurate information [14,15]. A recent literature review has reported that online social platforms could positively affect anatomy education, with YouTube being the most investigated platform followed by Facebook and Twitter [16]. One shared concern from the reviewed studies was the need to evaluate the educational value of YouTube videos [16]. Therefore, the aim of this study was to evaluate YouTube videos that demonstrated panoramic radiography in terms of preparatory and positioning procedures, and operator safety procedures. Following the evaluation, we identified high-quality videos that could serve as a reference for clinicians and dental students.

## 2. Materials and Methods

**Figure 1:**
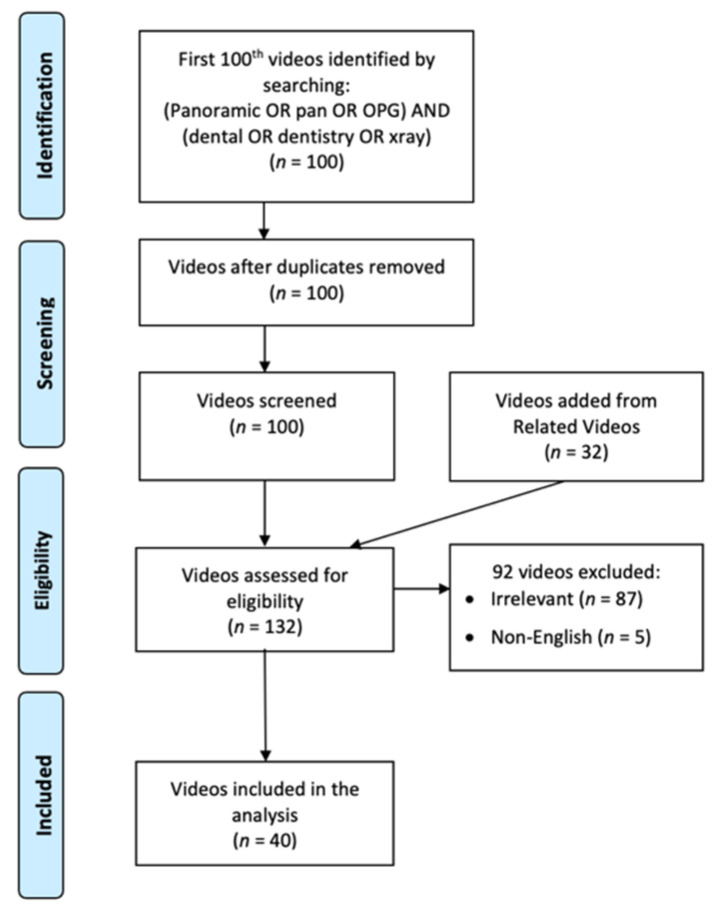
A flow chart showing the screening process of the YouTube videos.

A search for relevant YouTube videos was conducted in April 2022. The search string was: (panoramic OR pan OR OPG) AND (dental OR dentistry OR X-ray). The first 100 videos that resulted from the search and their related videos (recommended by YouTube as a list on the right of the screen) were screened. It should be noted that YouTube considers ranking of title, descriptions and video match to the query terms, engagement with that query, and watch-time, so that the ranking is dynamic [17]. Exclusion criteria included irrelevance (e.g., no demonstration of panoramic radiography procedures) and non-English videos. The entire video-screening process is illustrated by Figure 1. Finally, 40 videos were included and analyzed. For each included video, the following parameters were recorded: image receptor type, patient age, patient type (real patient, animation, or phantom head), patient preparation procedures, machine preparation, patient positioning, and operator safety.

The number of views, comments, likes, and channel subscribers were recorded, as well as the video duration and the age of the video. Pearson correlation tests were conducted to evaluate if any of these performance metrics were significantly correlated. A test was considered significant if *p* < 0.05.

Ethical approval was not applicable to this study.

## 3. Results

**Figure 2:**
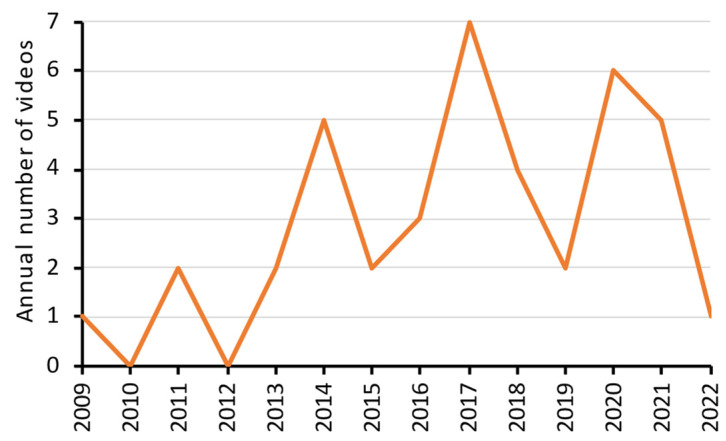
Number of YouTube videos uploaded each year that demonstrated panoramic radiography.

The details (including the web links) of the 40 videos on the demonstration of panoramic radiography are listed in Supplementary Table S1. The oldest one was uploaded on 22 July 2009, whereas the most recent one was uploaded on 30 January 2022. Year 2017 had the highest number of videos uploaded (*n* = 7). There was no obvious trend in the number of videos uploaded against the year (Figure 2).

Most of the videos demonstrated digital panoramic radiography with an adult patient (Table 1). Four videos involved a child patient. No video used a phantom head for the demonstration. For patient preparation procedures, most of the videos (95.0%) explained the procedures to the patient, but only 55.0% of the videos explicitly instructed the patient to remove (or explicitly showed the removal of) metallic objects from his/her head and neck region. One video even showed a patient wearing earrings during panoramic radiography, which were visualized in the resultant image (https://www.youtube.com/watch?v=ikqb-A5zZbs, accessed on 24 May 2022). Meanwhile, 70.0% of the videos had their patient wearing a lead apron during panoramic taking. For machine preparation, slightly over half (52.5%) of the videos showed the selection of imaging parameters, and 67.5% of them covered the bite-peg with a plastic barrier for infection control.

For patient positioning procedures, most of the videos (>92.5%) were consistent in terms of keeping the patient’s cervical spine straight, holding handles, biting incisors onto the bite-peg in edge-to-edge position, putting the chin on the chin rest, and stabilizing the head with head clamps (Table 2). Three videos demonstrated the procedure with machines that were designed without a chin rest (2 Sirona devices and 1 Gendex device). Meanwhile, the only video without using a head clamp showed small but consistent head movement of a child patient, implying the importance of head clamps for head stabilization (https://www.youtube.com/watch?v=JEgAjyPd9VQ, accessed on 24 May 2022). Meanwhile, only 60% of the videos instructed the patient to press his/her tongue onto the hard palate to eliminate the glossopharyngeal air space in the resultant radiographic image. Only 65.0% of the videos demonstrated the alignment of the beam marker (the canine light) in the canine region.

For operator safety procedures, none of the videos clearly showed that the operator was pressing the exposure button outside the radiographic room with its door closed (Table 3). Most of the videos either showed that the operator was pressing the button at the corridor outside the radiographic room/area with an open door, with no door, or without enough information on whether the door was closed.

Finally, a summary of the viewing metrics of the videos is listed in Table 4. On average, each video was viewed 61,318 times. It should be noted that one particular video (https://www.youtube.com/watch?v=cK726D70Sfg, accessed on 24 May 2022) has accumulated over 1.8 million views. It is a self-narrated video about a child visiting a hospital to have “dental X-rays” performed, which included ample time on a demonstration of panoramic radiography and a brief showing of cephalography. Each video had approximately 11 comments and 173 likes on average, and had a mean duration of 6–7 min. The latest video was uploaded in January 2022, whereas the oldest video was uploaded in July 2009. Interestingly, the two videos with film-based panoramic radiography were uploaded in 2011 and 2019, respectively. View count, comment count, and channel subscriber count were found to be positively correlated with like count (Table 5).

## 4. Discussion

This YouTube video survey has identified 40 videos that demonstrated panoramic radiography. Most of them involved a real, adult patient positioned within a panoramic machine that output an image in digital format. There was a wide range of variations in terms of patient and machine preparations, patient positioning, and operator safety procedures.

A recent multi-institutional survey found that 95% of responding undergraduates considered “YouTube videos on clinical procedures to be a helpful learning tool”, and 81% of them were very likely/likely to refer to a YouTube video to prepare for a clinical procedure that one has never attempted before [18]. Watching YouTube videos for educational purposes is not limited to students from a few developed countries. Instead, it is a global phenomenon [19]. Hence, the content quality of relevant videos should be evaluated, so that teachers and students could establish a customized watchlist for their educational/learning purposes. In panoramic radiography, for example, one should select videos showing a similar workflow with his/her own institution. One obvious example is patient positioning. Some panoramic machines are designed to operate without a chin rest (e.g., Sirona Orthophos), whereas some are compatible with patients biting a block of plastic foam-like disposable mouthpiece (or disposable bite-guides) that can replace a bite-peg (e.g., Morita Veraviewepocs). Choosing a compatible video would avoid confusion. This issue was clearly reported by students from a previous study on YouTube videos of endodontics: “…I guess like the steps are the same, but just the system used at school is different. It doesn’t completely match up” [10].

Besides workflow, infection control is another concern, especially during the COVID-19 pandemic. It was suggested that “the minimal personal protective equipment (PPE) for dental radiology staff should include disposable surgical mask, cap, gloves, long-sleeved gown, and goggles/face shield” and that “all non-critical items should be barrier-protected with plastic sheets or wraps, and should be changed after each patient” [20]. One should be very aware of the fact that some videos do not adhere to these latest recommendations. Three of the forty videos showed an operator with no mask, cap, gloves, and googles/face shield, and a patient biting directly onto the bite-peg without a plastic barrier (https://www.youtube.com/watch?v=JGa-qSkKA6s, accessed on 24 May 2022 and https://www.youtube.com/watch?v=ikqb-A5zZbs, accessed on 24 May 2022 (with the operator wearing a short-sleeved clinical uniform), and https://www.youtube.com/watch?v=AGqrEXbBmj8, accessed on 24 May 2022 (with the operator wearing a long-sleeved clinical uniform)). These three videos collectively accumulated 145,873 views and 364 likes. Junior students would be particularly affected if they had inadequate learning and experience with infection control concepts. It was reported that viral particles could be found on both the inner and outer surfaces of a surgical mask when a patient with COVID-19 coughed, whereas they were limited to the inner surface of N95 and KF94 masks [21]. This indicated that surgical masks might be less effective than the latter two and other respirators in filtering or containing COVID-19 viral particles. It is possible for patients to cough during dental radiographic procedures (though more likely for intraoral radiography), and therefore operators are strongly recommended to follow the recommendations of face coverage with at least a surgical mask.

The use of lead aprons in panoramic radiography is controversial. Here, 70% of the surveyed videos showed a patient wearing a lead apron during panoramic radiography. In fact, the International Atomic Energy Agency (IAEA), European Commission, the Faculty of General Dental Practice of the United Kingdom (FGDP), the American Academy of Oral and Maxillofacial Radiology (AAOMR), and the Japanese Society of Oral and Maxillofacial Radiology (JSOMFR) all agreed that the routine use of lead aprons for dental radiography and even cone-beam computed tomography is unnecessary, as it provides minimal additional benefit to the patient and might interfere with proper exposure if improperly designed or worn [22,23,24,25,26].

Having said that, lead aprons can be helpful in situations when other imaging parameters are not optimized, such as the use of analogue films of older generations that inherently require a larger radiation dose. Additionally, patients who are pregnant can be better reassured by wearing a lead apron. The concept of radiation protection keeps evolving [27]. Similarly, the teaching on the use of lead aprons and their application during clinical practice have also evolved from the past and will continue to evolve in the future.

Regardless, one very important aspect of the use of lead aprons during the COVID-19 pandemic is their disinfection with low-level disinfectants, such as quaternary ammonium compounds [20]. This, together with the disinfection of the panoramic machine, was not demonstrated in any of the videos. Dental teachers and students should be reminded of the importance of equipment disinfection to minimize disease transmission. Considering all parameters analyzed in this study, one good example that could be particularly useful for student learning is this video (7 min 14 s long, 75,953 views, 539 likes, posted 4.8 years ago): https://www.youtube.com/watch?v=6DcP3bGl9M8, accessed on 24 May 2022. Readers should be aware that the video did not demonstrate the selection of imaging parameters nor the use of a plastic barrier to cover the bite-peg, and the resultant panoramic image showed a relatively flat occlusal curve that implied non-ideal head positioning (Frankfort plane inclined instead of being horizontal).

This survey has some limitations. First, only videos on YouTube were considered. Second, only videos narrated or voiced in English were evaluated. Besides, it was not possible to search for and evaluate private or unlisted videos on YouTube. Meanwhile, qualitative analysis was not performed to evaluate the comments posted for the videos. Unfortunately, YouTube has removed the dislike count. Hence, it was not possible to record these data. In the long run, it would be beneficial for dental schools to produce their own customized instructional videos for various dental radiology procedures, besides panoramic radiography. Of course, such video production could be challenging for dental staff, and it requires ample support regarding equipment and technical expertise from the information technology department. As a potential alternative, dental schools can create a curated playlist of free videos that are suitable for their educational purposes. Either way, students can gain access to a list of trusted or proven videos to minimize their exposure to videos with irrelevant information or misinformation.

## 5. Conclusions

This YouTube video survey identified 40 videos on panoramic radiography demonstrations. Most of the videos demonstrated digital panoramic radiography with an adult patient. Procedures on patient and machine preparations as well as patient positioning were generally explained well. However, most videos did not well-demonstrate operator safety details concerning the use of adequate personal protective equipment. It is recommended that videos produced in the future should show operators wearing full PPE comprising of a head cap, eye protection, a surgical mask, gloves, and a long-sleeved disposable gown. Future videos should also show demonstrations or mention features tailored to child patients, such as the use of “child mode” to reduce radiation dose, or the availability of a fast scan for certain panoramic machine models. Clinicians and teaching staff should carefully critique the content of such instructional videos and refer to the contents from other sources such as user manuals and latest recommendations from local authorities. Teaching staff should recognize that YouTube is already being used as a learning resource, and therefore they have an opportunity to provide context for or curate what students may be accessing. Future studies should similarly evaluate the utility of YouTube videos for demonstrating other clinical procedures in healthcare education.

## Figures and Tables

**Table 1:** Frequency counts of (A) image receptor type, (B) patient type, (C) patient preparation, and (D) machine preparation procedures.

Procedure	No. of Videos	% (of 40)
(A) Image receptor type		
Digital	38	95.0
Film	2	5.0
(B1) Patient age		
Adult	35	87.5
Child	4	10.0
Teenager	1	2.5
(B2) Patient type		
Real patient	36	90.0
Animation	4	10.0
Phantom head	0	0
(C1) Explicitly removed or instructed to remove metallic objects from head and neck		
Yes	22	55.0
No	18	45.0
(C2) Explain the procedure		
Yes	38	95.0
No	2	5.0
(C3) Patient wearing lead apron		
Yes	28	70.0
No	11	27.5
N/A	1	2.5
(D1) Selected imaging parameters (mode, body size, etc.)		
Yes	21	52.5
No	19	47.5
(D2) Used plastic barrier to cover the bite-peg		
Yes	27	67.5
No	10	25.0
N/A	3	7.5

N/A, not applicable or could not be determined.

**Table 2:** Frequency counts of patient positioning procedures.

Procedure	No. of Videos	% (of 40)
(1) Straight cervical spine		
Yes	38	95.0
No	2	5.0
(2) Holding handles		
Yes	39	92.5
N/A	1	2.5
(3) Incisors biting edge-to-edge onto the bite-peg		
Yes	38	95.0
N/A	2	5.0
(4) Chin on chin rest		
Yes	37	92.5
N/A	3	7.5
(5) Using head clamps		
Yes	39	97.5
No	1	2.5
(6) Using beam marker to align mid-sagittal plane		
Yes	33	82.5
No	3	7.5
N/A	4	10.0
(7) Using beam marker to align Frankfort plane		
Yes	31	77.5
No	5	12.5
N/A	4	10.0
(8) Using beam marker to align canine region		
Yes	26	65.0
No	9	22.5
N/A	5	12.5
(9) Instructed to press tongue onto the hard palate		
Yes	24	60.0
No	15	37.5
N/A	1	2.5

N/A, not applicable or could not be determined.

**Table 3:** Frequency counts of operator safety procedures.

Procedure	No. of Videos	% (of 40)
(1) Operator position		
Totally shielded with door closed	0	0
Shielded position with door opened (or no scene showing door closure)	28	70.0
Inside the room with lead apron	1	2.5
Totally unprotected	1	2.5
N/A	10	25.0
(2) Wearing head cap		
Yes	5	12.5
No	25	62.5
N/A	10	25.0
(3) Wearing eye protection or face shield		
Yes	5	12.5
No	25	62.5
N/A	10	25.0
(4) Wearing surgical mask		
Yes	11	27.5
No	19	47.5
N/A	10	25.0
(5) Wearing gloves		
Yes	18	45.0
No	17	42.5
N/A	5	12.5
(6) Body wear		
Disposable gown	6	15.0
Clinical uniform	16	40.0
Lab coat	6	15.0
Casual wear	4	10.0
N/A	8	20.0

N/A, not applicable or could not be determined.

**Table 4:** Viewing metrics of the 40 videos.

Metric	Mean ± SD	Min; Max
View count	61,318 ± 288,383	26; 1,835,204
Comment count	11.3 ± 33.1	0; 178
Like count	173.3 ± 601.1	0; 3800
Duration (s)	381.5 ± 409.7	67; 1979
Channel subscriber count	15,242 ± 50,885	2; 276,000
Age of video (years)	4.9 ± 3.2	0.3; 12.8

**Table 5:** Pearson correlation between the viewing metrics.

Metric	Comment Count	Like Count	Duration (s)	Channel Subscriber Count	Video Age (Years)
View count	0.178 (*p* = 0.330)	0.985 (***p* < 0.001**)	−0.050 (*p* = 0.760)	0.331 (*p* = 0.052)	0.147 (*p* = 0.365)
Comment count		0.666 (***p* < 0.001**)	0.230 (*p* = 0.206)	0.332 (*p* = 0.073)	−0.280 (*p* = 0.121)
Like count			−0.038 (*p* = 0.816)	0.418 (***p* = 0.012**)	0.086 (*p* = 0.596)
Duration (s)				0.077 (*p* = 0.662)	0.096 (*p* = 0.558)
Channel subscriber count					−0.188 (*p* = 0.280)

*p* values are bolded if <0.05.

## Data Availability

All data are available in the manuscript.

## Supplementary Materials

The following supporting information can be downloaded at: https://www.mdpi.com/article/10.3390/healthcare10061093/s1, Table S1: Details of the 40 videos on the demonstration of panoramic radiography.
